# WiMonitor: Continuous Long-Term Human Vitality Monitoring Using Commodity Wi-Fi Devices

**DOI:** 10.3390/s21030751

**Published:** 2021-01-22

**Authors:** Xiaopeng Niu, Shengjie Li, Yue Zhang, Zhaopeng Liu, Dan Wu, Rahul C. Shah, Cagri Tanriover, Hong Lu, Daqing Zhang

**Affiliations:** 1 Key Laboratory of High Confidence Software Technologies (Ministry of Education), School of Electronics Engineering and Computer Science, Peking University, Beijing 100871, China; nxpeng@pku.edu.cn (X.N.); lishengjie@pku.edu.cn (S.L.); zy.zhang@pku.edu.cn (Y.Z.); liuzp@pku.edu.cn (Z.L.); dan@pku.edu.cn (D.W.); 2 Intel Corporation, Santa Clara, CA 95054, USA; rahul.c.shah@intel.com (R.C.S.); hong.lu@intel.com (H.L.); 3 Intel Corporation, Hillsboro, OR 97124, USA; cagri.tanriover@intel.com; 4 Télécom SudPais, Institut Polytechnique de Paris, 91764 Palaiseau, France

**Keywords:** Wi-Fi, device-free, long-term human vitality, elderly care

## Abstract

For a large population of elderly who live alone, a continuous long-term daily monitoring system is critical and imminently needed to enhance the quality of their lives. Continuous monitoring of vitality information (i.e., which area the elder is staying in, the motion state and activity intensity of an elder) is essential for elderly care. In this paper, we use existing commodity Wi-Fi devices to design and implement a long-term device-free human daily vitality system, WiMonitor. Our system can continuously capture the target’s vitality information in a multi-room home environment without compromising the privacy of the target. In a continuous 22-day experiment, WiMonitor successfully captures the human vitality information accurately. We believe our system can provide valuable long-term monitoring data for both researchers and health care personnel.

## 1. Introduction

Population aging is a global issue affecting most countries. In 2019, there were 703 million persons aged 65 years or over in the global population, and this number is projected to double to 1.5 billion in 2050 [1]. By 2050, one in six people in the world will be over age 65, and roughly 90 percent of that population prefers to stay in their home rather than in a nursing home [2,3]. In Austria, 51.4% of all households of people aged older than 65 years were single-households in 2018. In Europe, more than a third of older people live alone. In the US, nearly 29% of the 46 million community-dwelling older adults live alone [4,5].

Elderly people who live alone can develop poor health, they may have an accident at home and have no way of alerting someone that they need help [6]. Thus, a continuous long-term daily monitoring system is critical and imminently needed for elderly health care. Specifically, daily life routine and abnormality detection are key for good elderly health care. Besides, regular daily physical activity is one of the most important things people can do to improve their health, and such daily physical activity can decrease the risk of chronic diseases [7]. According to the energy consumed as people perform certain activities, daily activities can be divided into different levels [8,9]. Activity levels may remain relative stable over weeks or months, an early and accurate awareness of decreasing activity levels can act as a warning sign to trigger early intervention [10]. High-level semantics such as the area staying rate of the target, the motion state of the target and long-term physical activity intensity levels are important health-related information. Moreover, if we can continuously and non-intrusively capture these health-related daily life information of elders over the scale of weeks and months, then that would provide a valuable long-term monitoring system for health care.

However, capturing human daily life information for a long period of time is challenging. An obvious solution is deploying cameras in each room to monitor all the areas and record all activities an elder performs [11,12]. However, camera-based solutions are limited by line-of-sight and pose severe privacy concerns. Besides camera-based methods, researchers have also explored wearable-based methods [13,14] for indoor human monitoring in the past few years. However, wearable-based solutions are able to sense the motion of local body parts but cannot obtain the whole body state and provide location information. Moreover, people tend to forget or are reluctant to wear wearable devices [15]. An ideal long-term human daily life sensing system at home should be non-intrusive, does not sacrifice the privacy of the target and is at a low cost.

Wi-Fi is particularly promising as Wi-Fi access points and devices are already ubiquitous and thus there is no need of deploying any extra infrastructure. The latest research explores the possibility of employing Wi-Fi channel state information (CSI) for indoor localization [16,17,18,19,20,21,22], gesture/activity recognition [23,24], gait recognition [25] and motion detection [26,27,28,29,30,31]. Current Wi-Fi based activity recognition systems usually aim to identify specific activities and require manually segmenting the activities in advance. Current Wi-Fi-based localization systems require labor-intensive training, phase calibration, and the 90th percentile error of these methods can be up to 2 m [19], thus these systems can cause lots of false results when performing area detection. Moreover, we find that, in a lot of scenarios in smart home environment, for elder daily life monitoring, obtaining continuous health-related information (i.e., elder’s staying area, motion state and activity intensity levels) during a specific time period is essential. Thus, the area where the elderly stays in, the motion state (still or non-still [28]) and the activity intensity level of the elderly are three important health-related information for a continuous long-term elderly monitoring system. 

In this paper, we introduce the notion of vitality to represent the statistical measure of one’s staying area (room-level), motion state and activity intensity levels during a specific time period (such as 24 h). Through statistical analysis of vitality information, we can reveal the daily life routine of the elder and find any abnormalities. For example, if the elder stays too long in the toilet in the still state which is different from his/her usual daily life routine, then that may indicate an abnormality calling for attention [28]. 

There has been prior research work on human vitality sensing—WiVit system [28]. However, it cannot estimate activity intensity of the target, it utilized Doppler-Music method to extract Doppler frequency shift from conjugate multiplication between the CSI of two antennas, and used the power of Doppler frequency shift to detect human motion. Since the operation of conjugate multiplication amplifies the amplitude noise, when user performs small-scale motion actions (such as turning over on the bed), it may be difficult to discriminate the small-scale motion actions from environmental noises, thus could not effectively detect such small-scale motion. In this paper, we propose methodologies of robust long-term area detection, motion detection and activity intensity estimation. We implement a system—WiMonitor, which can continuously monitor human vitality information for a long period of time in a typical smart home environment.

The main contributions of this paper are as follows:

- We propose a method to achieve robust long-term room-level area detection.
- We design a metric to estimate the intensity of physical activity and explain the metric’s capability for activity intensity estimation.
- We implement and deploy a real-time Human Vitality Monitor System—WiMonitor, using commodity WiFi devices for continuous long-term monitoring in a smart home environment, and ask four volunteers to live in the smart home for 24 h in a continuous 22-day experiment. The long-term experiment demonstrates that WiMonitor is able to accurately reveal the daily life routines of different volunteers. The experiment shows that the system can be used to infer the routine habits of people, detect any abnormality. We believe this non-intrusive sensing system and sensing data generated make long-term elderly care and health support at home viable.

The rest of this paper is organized as follows. Section 2 introduces the related work. Section 3 introduces the preliminaries of CSI and other related knowledge. Section 4 presents the methodologies of proposed system in detail. Section 5 presents the experimental results analysis. Section 6 discusses the existing indoor positioning techniques and compares with ours. Section 7 draws the conclusion of this work.

## 2. Related Work

In recent years, with the rapid development of sensing technology in the era of the Internet of Things (IOT), a variety of human sensing technologies have been developed, including wearable sensors [13,14,32], camera [11,12], radio frequency (e.g., WiFi) [8,16,17,18,19,20,21,22,23,24,25,26,27,28,29,30], acoustic-based sensors [33,34], infrared [35,36] and so forth. wearable sensor-based technologies require users to wear a specialized device for sensing, which is inconvenient for users. Camera-based technologies can only work well under good lighting conditions and may bring privacy concerns. Acoustic-based technologies are vulnerable to ambient noise and the sensing area is limited. Infrared-based technologies need dedicated infrastructure and are easy to block by obstacles. WiFi-based technologies reuse WiFi devices, and have the characteristics of low cost, easy to deploy, privacy preservation and non-intrusive. These characteristics enable WiFi-based technologies draw more attention among researchers. In this paper, we focus on long-term human vitality monitoring in a multi-room smart home environment using the commodity Wi-Fi devices. Our monitoring system not only could detect which area the user is staying in, but also could monitor the user’s motion state and activity intensity. We also discuss existing research most relevant to our work.

### 2.1. WiFi-Based Device-Free Indoor Localization

The existing device-free indoor localization systems can be categorized as fingerprint-based and geometric mapping-based. Dang et al. [25] uses the post-processed CSI amplitude and phase as fingerprint data to build the fingerprint database. Xiao et al. [37] use the distribution of the CSI amplitudes over the all sub-carriers and the multiple antennas as fingerprint of corresponding positions, in order to improve the localization accuracy. Most of fingerprint-based systems are difficult to deploy since these systems need labor intensive offline training and are sensitive to the changes of the environment. Unlike fingerprint-based method, geometric mapping-based method does not need to build fingerprint database. MaTrack [17] employ the angle-of-arrival (AoA) information to locate the user. However, MaTrack needs phase calibration due to random phase offset of two antennas. Indotrack [18] applies conjugate multiplication between the CSI of two antennas to remove the random phase offset and achieves better localization performance than MaTrack. Through these works can get better localization performance, they cannot obtain accurate area information at room-level. 

In a typical home environment, room-level localization is sufficient for a lot of real-life scenarios. Obtaining the accurate area information (i.e., which room the user is staying in) is much more useful than knowing inaccurate fine-grained location information for elder daily life monitoring. WiVit [28] applies a Doppler-Music method [18] to extract the path length change speeds, and utilizes the relationship between path length change speed and the user’s position to calculate which area the user is located. However, this method is not robust enough which needs calibration when environment changes. Wiborder [16] applies conjugate multiplication between the CSI of two antennas to construct parameters to discriminate the sensing boundaries which formed by walls of different areas. Wiborder is the first work through boundary sensing determination to achieve accurate area detection. However, CSI amplitude contains noise due to the noise of Automatic Gain Controller (AGC) and the environmental noise, conjugate multiplication between the CSI of two antennas could amplify the amplitude noise, thus the sensing boundary parameter is not robust to achieve long-term area detection. In this paper, we propose a method to remove the noise of AGC on CSI amplitude to get more stable sensing boundary parameter and achieve long-term area detection.

### 2.2. WiFi-Based Device-Free Human Motion Detection

As human motion detection plays an important role in many smart home applications such as intrusion detection and activity recognition, a lot of device-free human motion detection systems have been developed using WiFi CSI. Dong et al. [26] extracted both the time and frequent domain features from CSI amplitude to detect human motion in both flat floor and staircase settings. MoSense [27] utilizes the variance of phase difference for human motion detection. The key idea is that the phase difference remains stable when there is no motion, and changes when the user is moving. These research works require offline training and calibration, and the threshold value which is used to distinguish the still and motion status of the target varies in different environments. There are also have some research works which do not require training phase, WiVit [28] utilizes Doppler-Music method to obtain Doppler frequency spectrum and uses the power of Doppler frequency spectrum as the feature to detect human motion. However, both the power of CSI and Doppler-Music method could amplify the amplitude noise, when the target is far from transceivers, the amplitude noise could mask the small CSI changes caused by user’s small-scale motion. WiDetect [31] uses the auto correlation function of the power of CSI for motion detection.

## 3. Preliminaries

In this section, we first introduce the CSI Primer, and then introduce two novel technologies which can reduce the noises of raw CSI signals.

### 3.1. CSI Primer

In the indoor environment, WiFi signals propagate from transmitter to receiver through multiple paths, i.e., one Line-of-Sight (LOS) path, and multiple paths from surrounding objects such as walls, furniture and the human body. The signal arriving at the receiver is a superposition of signals from all the paths, this phenomenon is called multipath effect [28,38]. The wireless propagation channel can be modeled as Channel Impulse Response (CIR) in time domain to characterize the individual paths, CIR *h*(*τ*) can be denoted as:

(1) $$h\left(\tau \right)=\sum_{i=1}^{N}a_{i}e^{-j\theta_{i}}\delta \left(\tau -\tau_{i}\right),$$

where *a_i_*, *θ_i_* and *τ_i_* represent the amplitude, phase and time delay of the *i*th path, respectively. *N* is the total number of multi-path, and *δ*(*τ*) is the Dirac delta function [39]. Channel frequency response (CFR) is the Fourier transform of CIR, characterizes the small-scale multipath effect and the combined effect of scattering, reflecting and fading with distance. Let *X*(*f*,*t*) and *Y*(*f*,*t*) represent the frequency domain representations of transmitted and received signals, respectively. CFR for a carrier frequency *f* at time *t. H*(*f*,*t*) can be represented as:

(2) Y(f,t)=H(f,t)×X(f,t)+N,

where *N* represents the Gaussian noise [24]. In WiFi 802.11n, CSI is the sampled version of CFR at subcarrier level. In the area of Wi-Fi sensing, the ideal CSI can roughly be denoted as:

(3) $$H\left(f,t\right)=\sum_{i=1}^{L}\alpha_{i}e^{-j2\pi \frac{d_{i}\left(t\right)}{\lambda }},$$

where *α_i_* is the complex attenuation, *d_i_*(*t*) is the propagation length of the *i*th path, *λ* is the wavelength and *L* is the total number of multi-paths.

The multiple paths can be divided into static paths and dynamic paths [24]. The static paths are composed of the LoS path and the reflected paths from the static objects in the environment, which do not change with time. While the dynamic paths are the signal paths induced by the moving targets. Thus, the CSI can be rewritten as:

(4) $$H\left(f,t\right)=H_{s}\left(f,t\right)+H_{d}\left(f,t\right)=Se^{-j\varphi }+\sum_{k=1}^{K}\alpha_{k}e^{-j2\pi \frac{d_{k}\left(t\right)}{\lambda }},$$

where *H_s_*(*f*,*t*) is the static component which is the sum of CFRs for static paths, and *S* is the attenuation, *φ* is the phase shift of the static component. *H_d_*(*f*,*t*) is the dynamic component which is the sum of CFRs for dynamic paths, and *α_k_* is the complex attenuation, *d_k_*(*t*) is the path length of the *k*th path, respectively. 

However, the CSI measurements provided by commercial WiFi devices contain two major types of noise: the amplitude noise and the phase noise [38]. Due to the power amplifier uncertainty of AGC and environmental noise, the CSI amplitude has noise Δ*_AGC_* and *ε*. Since the transmitter and receiver are not time-synchronized, there is a time-varying random phase offset $e^{-j\theta_{offset}}$ in each CSI sample [16,18]. Considering these noises, the CSI can be rewritten as:

(5) $$\begin{array}{ll} H^{\prime }\left(f,t\right) & =\Delta_{AGC}e^{-j\theta_{offset}}\left(H\left(f,t\right)+\varepsilon \right)\\ & =\Delta_{AGC}e^{-j\theta_{offset}}\left(Se^{-j\varphi }+\sum_{k=1}^{K}\alpha_{k}e^{-j2\pi \frac{d_{k}\left(t\right)}{\lambda }}+\varepsilon \right)\\ & \approx \Delta_{AGC}e^{-j\theta_{offset}}(Se^{-j\varphi }+\sum_{k=1}^{K}\alpha_{k}e^{-j2\pi \frac{d_{k}\left(t\right)}{\lambda }}), \end{array}$$

This CSI noise prevents us from achieving satisfactory sensing performance. In order to remove such noise, several signal processing technologies have been proposed. Among these technologies, CSI conjugate multiplication and CSI ratio are two novel technologies which have been widely used in existing research works [16,18,28,40,41].

### 3.2. CSI Conjugate Multiplication

A commodity WiFi card usually has multiple antennas, the time-varying random phase offsets are the same across different antennas on a WiFi card as they share the same RF oscillator [18]. Thus, conjugate multiplication between the CSI of two antennas can remove the time-varying random phase offsets [18].

(6) $$\begin{array}{ll} H_{cm}\left(f,t\right) & =H_{1}{}^{\prime }\left(f,t\right)\bar{H_{2}{}^{\prime }\left(f,t\right)}\\ & \approx \left(\Delta_{AGC}e^{-j\theta_{offset}}\left(S_{1}e^{-j\varphi_{1}}+\sum_{k1=1}^{K1}\alpha_{k1}e^{-j2\pi \frac{d_{k}{}_{1}\left(t\right)}{\lambda }}\right)\right)\left(\Delta_{AGC}e^{j\theta_{offset}}\left(S_{2}e^{j\varphi_{2}}+\sum_{k2=1}^{K2}\alpha_{k2}e^{j2\pi \frac{d_{k}{}_{2}\left(t\right)}{\lambda }}\right)\right)\\ & =\Delta_{AGC}{}^{2}\left(S_{1}e^{-j\varphi_{1}}+\sum_{k1=1}^{K1}\alpha_{k1}e^{-j2\pi \frac{d_{k}{}_{1}\left(t\right)}{\lambda }}\right)\left(S_{2}e^{j\varphi_{2}}+\sum_{k2=1}^{K2}\alpha_{k2}e^{j2\pi \frac{d_{k}{}_{2}\left(t\right)}{\lambda }}\right)\\ & =\Delta_{AGC}{}^{2}(\underset{①}{\underbrace{S_{1}S_{2}e^{j\left(\varphi_{2}-\varphi_{1}\right)}}}+\underset{②}{\underbrace{S_{1}e^{-j\varphi_{1}}\sum_{k2=1}^{K2}\alpha_{k2}e^{j2\pi \frac{d_{k}{}_{2}\left(t\right)}{\lambda }}}}\\ & \;+\underset{③}{\underbrace{S_{2}e^{j\varphi_{2}}\sum_{k1=1}^{K1}\alpha_{k1}e^{-j2\pi \frac{d_{k}{}_{1}\left(t\right)}{\lambda }}}}+\underset{④}{\underbrace{\sum_{k1=1}^{K1}\alpha_{k1}e^{-j2\pi \frac{d_{k}{}_{1}\left(t\right)}{\lambda }}\sum_{k2=1}^{K2}\alpha_{k2}e^{j2\pi \frac{d_{k}{}_{2}\left(t\right)}{\lambda }}}}), \end{array}$$

The conjugate multiplication between the CSI of two antennas can be denoted as Equation (6), where *H_cm_*(*f*,*t*) is the output of conjugate multiplication, $H_{1}^{\prime }\left(f,t\right)$ is the CSI of the first antenna, $H_{2}^{\prime }\left(f,t\right)$ is the conjugation of the CSI of the second antenna. 

In Equation (6), the term ① is the product of the static components and can be treated as a constant within a short time period. The term ② and term ③ are two products of static component of one antenna and dynamic component of another antenna. The term ④ is the product of dynamic components which can be ignored as the value is very small. From Equation (6), we can see that the random phase offset has been removed from CSI. However, conjugate multiplication between the CSI of two antennas amplifies the amplitude noise. 

### 3.3. CSI Ratio

Assuming there is only one reflection path corresponding to the human target’s movement, the dynamic component is the path reflected from the human target while the static component is composed of the LoS propagation and other reflection paths from static objects in the environment. The CSI Ratio is calculated by taking the division operation of CSI between two antennas, which is defined as follows:

(7) $$\begin{array}{ll} H_{r}\left(f,t\right) & =\frac{H_{1}{}^{\prime }\left(f,t\right)}{H_{2}{}^{\prime }\left(f,t\right)}\\ & \approx \frac{\Delta_{AGC}e^{-j\theta_{offset}}\left(S_{1}e^{-j\varphi_{1}}+\alpha_{1}e^{-j2\pi \frac{d_{1}\left(t\right)}{\lambda }}\right)}{\Delta_{AGC}e^{-j\theta_{offset}}\left(S_{2}e^{-j\varphi_{2}}+\alpha_{2}e^{-j2\pi \frac{d_{2}\left(t\right)}{\lambda }}\right)}\\ & =\frac{S_{1}e^{-j\varphi_{1}}+\alpha_{1}e^{-j2\pi \frac{d_{1}\left(t\right)}{\lambda }}}{S_{2}e^{-j\varphi_{2}}+\alpha_{2}e^{-j2\pi \frac{d_{2}\left(t\right)}{\lambda }}}\\ & =\frac{S_{1}e^{-j\varphi_{1}}+\alpha_{1}e^{-j2\pi \frac{d_{1}\left(t\right)}{\lambda }}}{S_{2}e^{-j\varphi_{2}}+\alpha_{2}e^{-j2\pi \frac{d_{1}\left(t\right)}{\lambda }}e^{-j2\pi \frac{\Delta d}{\lambda }}} \end{array}$$

where $H_{1}^{\prime }\left(f,t\right)$, $H_{2}^{\prime }\left(f,t\right)$ are the CSI of the first antenna and the second antenna, respectively. Through division operation, we can see that the CSI amplitude noises and the random phase offset are both removed. Let *a* = *α*_1_, *b* = $S_{1}e^{-}{}^{j\varphi_{1}}$, *c =* $\alpha_{2}e^{-j2\pi \frac{\Delta d}{\lambda }}$, *d* = $S_{2}e^{-}{}^{j\varphi_{2}}$, *z =* $e^{-j2\pi \frac{d_{1}\left(t\right)}{\lambda }}$. The CSI ratio can be rewritten as follows:

(8) $$H_{r}\left(f,t\right)=\frac{az+b}{cz+d},$$

Since the parameters *a*, *b*, *c*, *d* are all constant complex numbers, z represents a circle in complex plane, its phase represents the dynamic reflected path. Thus, the CSI Ratio can be regarded as the form of Mobius transformation [40].

The result of CSI Ratio is still a complex number with amplitude and phase, the amplitude is the ratio of the raw CSI amplitudes and the phase is the phase difference of the raw CSI phases. It has been proved in [40,41] that the CSI Ratio has the follow properties:

- CSI Ratio has higher signal-to-noise-ratio(SNR) than raw CSI and CSI conjugate multiplication, and changes following a circular pattern in complex plan;
- If the reflection path length change is exactly one wavelength, the CSI ratio will rotate exactly 2π in complex plane;
- If the reflection path length increases, CSI ratio rotates clockwise, otherwise, it rotates counterclockwise.

## 4. Methodologies

Since WiMonitor is a long-term human vitality monitoring system, it contains two major components: long-term area detection and long-term activity intensity estimation (including motion detection). As for area detection, Wiborder [16] constructed a boundary sensing parameter and depicted the area-transition-diagram for multi-room area detection. The sensing boundary parameter is the Rayleigh fitting parameter of the conjugate multiplication between the CSI of two antennas within a short time window, it reflects the fluctuation of the amplitude of the conjugate multiplication between the CSI of two antennas. Due to the power amplifier uncertainty of AGC and environmental noises, the amplitude of the CSI measurements contains a relative large noise. The conjugate multiplication operation amplifies the amplitude noise, so the sensing boundary parameter of Wiborder is not robust enough for long-term monitoring. In this section, we first analyze the noise of AGC, and then introduce the proposed methodology for removing this noise. At last, we introduce the CSI metric for activity intensity estimation.

### 4.1. Remove the Noise Caused by AGC

**Figure 1:**
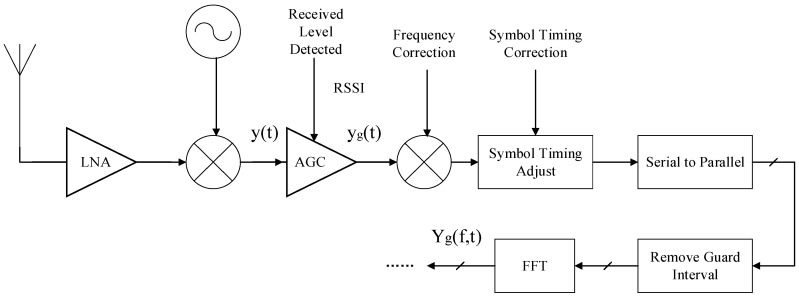
The signal processing in the Wi-Fi receiver block diagram.

Figure 1 illustrates the signal processing in the Wi-Fi receiver block diagram [42]. The signal from receiver antenna is down converted to base band signal *y*(*t*) through a mixer. Due to the path loss and multipath fading, the received signal strength is weaker than the transmitted signal strength. In order to maintain stable received signal strength for better wireless communication, AGC could amplify the signal strength dynamically according to Received Signal Strength Indicator (RSSI). The amplifier gain is large when the received signal strength is weak, so the amplifier gain can be considered as the function of RSSI δ(RSSI). Let *y_g_*(*t*) represents the received signal after AGC, *y_g_*(*t*) can be denoted as:

(9) $$y_{g}(t)=\delta \left(RSSI\right)\cdot y(t),$$

According to the properties of discrete Fourier transfer (DFT), the frequency domain of received signal after AGC *Y_g_*(*f*,*t*) can be denoted as:

(10) $$Y_{g}\left(f,t\right)=\delta \left(RSSI\right)\cdot Y\left(f,t\right),$$

where *Y*(*f*,*t*) is the frequency domain of received signal before AGC. According to Equation (2), the CSI after the AGC gain *H_g_*(*f*,*t*) can be denoted as:

(11) $$H_{g}\left(f,t\right)=\delta \left(RSSI\right)\cdot H\left(f,t\right),$$

As for wireless sensing, the CSI is modelled to describe the state of wireless channel, which is not include AGC, so *δ*(*RSSI*) is the noise which needs to be removed. However, the value of function *δ*(*RSSI*) is not accessible due to the hardware limitations of commodity WiFi card. Fortunately, through Intel 5300 CSI Tools [43], we can get RSSI and the gain coefficient of AGC agc in every received CSI packet. Since *agc* is related to *δ*(*RSSI*) and is controlled by RSSI, we can use the function *γ*(*agc*) instead of *δ*(*RSSI*) to remove the noise of AGC. 

Let *y_g_*(*n)* and *y*(*n)* be the sampled versions of *y_g_*(*t)* and *y*(*t)*, respectively. According to Parseval’s Theorem, we can get Equations (12) and (13):

(12) $$\sum_{n=0}^{N-1}{\left|y_{g}\left(n\right)\right|}^{2}=\frac{1}{N}\sum_{k=0}^{N-1}{\left|Y_{g}\left(k\right)\right|}^{2},$$

(13) $$\sum_{n=0}^{N-1}{\left|y\left(n\right)\right|}^{2}=\frac{1}{N}\sum_{k=0}^{N-1}{\left|Y\left(k\right)\right|}^{2},$$

where *N* is the number of samples. According to Equations (10), (12) and (13), we can get Equation (14):

(14) $$\gamma {\left(agc\right)}^{2}=\delta {\left(RSSI\right)}^{2}=\frac{1}{N}\cdot \frac{\sum_{k=0}^{N-1}{\left|Y_{g}\left(k\right)\right|}^{2}}{\sum_{n=0}^{N-1}{\left|y\left(n\right)\right|}^{2}},$$

The denominator of Equation (14) represents the received signal strength before AGC. Through Intel 5300 CSI Tools, the denominator can be calculated as:

(15) $$\sum_{n=0}^{N-1}{\left|y\left(n\right)\right|}^{2}=10^{\frac{RSSI-44-agc}{10}},$$

The numerator of Equation (14) represents the signal power after AGC in the frequency domain, and can be calculated as [42]:

(16) $$\sum_{k=0}^{N-1}{\left|Y_{g}\left(k\right)\right|}^{2}=\sum_{k=0}^{N-1}{\left|H_{g}\left(k\right)\cdot LTF\left(k\right)\right|}^{2}=\sum_{k=0}^{N-1}{\left|H_{g}\left(k\right)\right|}^{2},$$

where *LTF* is the Long Training Field, which is used to estimate the CSI [42]. According to Equations (14)–(16), the function *γ*(*agc*)^2^ can be denoted as:

(17) $$\gamma {\left(agc\right)}^{2}=\frac{1}{N}\cdot \frac{\sum_{k=0}^{N-1}{\left|H_{g}\left(k\right)\right|}^{2}}{10^{\frac{RSSI-44-agc}{10}}},$$

**Figure 2:**
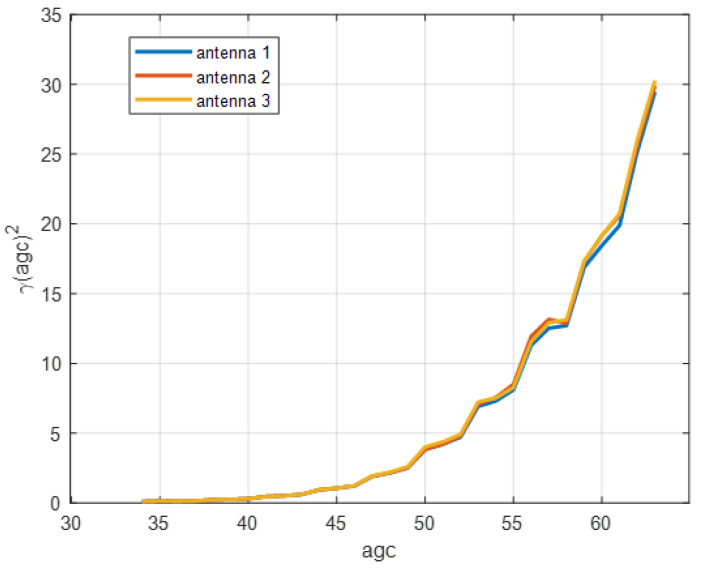
The relationship between $\gamma^{2}\left(agc\right)$ and agc.

In order to get the relationship of *γ*^2^(*agc*) and *agc*, we have collected a sufficiently large volume of CSI data. During data collection, we change the positions of transceivers as much as possible in order to get as many *agc* values as possible. Then, through Equation (17), we can obtain the value of *γ*^2^(*agc*) corresponding to each *agc*, and we can get the relationship of *γ*^2^(*agc*) and *agc*, which is shown in Figure 2. From Figure 2, we can see that the value of *γ*^2^(*agc*) increases with the increases of *agc*. That is to say, when the received signal power is small, agc will be increased in order to sustain a stable level of the received signal power. Moreover, the three antennas of an Intel 5300 WiFi Card use the same AGC gain circuit.

Thus, we can use Equation (18) to remove the noise of AGC:

(18) $$\left|H\left(f,t\right)\right|=\frac{\left|H_{g}\left(f,t\right)\right|}{\gamma \left(agc\right)},$$

**Figure 3:**
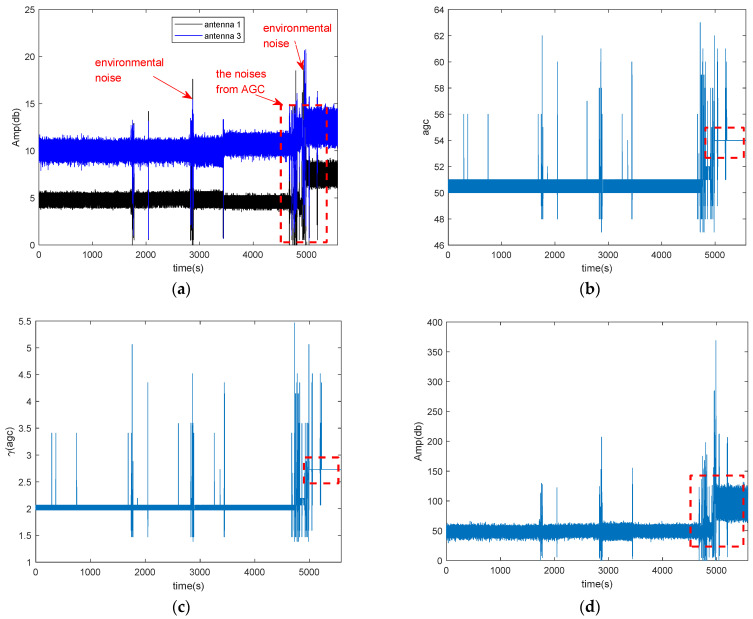
The impact of AGC and environmental noise on the amplitude of CSI in the silence environment. (**a**) CSI amplitude of two antennas; (**b**) the agc of the corresponded CSI amplitude; (**c**) the value of the function of agc; (**d**) the amplitude of conjugate multiplication between the CSI of two antennas.

Due to the noise of AGC, the CSI amplitude could fluctuate when there is no human motion or environment changes. So the thresholds for area detection of Wiborder will change even in the silence environment, thus Wiborder is not suitable for long-term area detection. In order to evaluate the effectiveness of our proposed method for remove the noise of AGC, we collect CSI data for a long time (about 1.5 h) in the silence environment. Figure 3 shows the impact of AGC and environmental noise on the amplitude.

Figure 3a shows the CSI amplitudes of two antennas, which contain the environmental noise and the noise of AGC. From Figure 3a, we can find besides some fluctuations which caused by environmental noise, the amplitude is divided into two segments at about 5000 s, as marked by red rectangle. Figure 3b,c shows the coefficients *agc* and *γ*(*agc*), respectively. There is also has a similar but smaller jump at around 3500 s in the Figure 3a, but the *agc* of the corresponding CSI amplitude remains relative stable except for some outliers. These outliers may be caused by environmental noise, how to remove the environment noise is out of scope of our paper. From Figure 3b,c, we can find that *agc* and *γ*(*agc*) are also divided into two segments at about 5000 s. Therefore, we can conclude that the jumping of amplitude at about 5000 s is caused by AGC. Figure 3d shows the amplitude of conjugate multiplication between the CSI of two antennas. From Figure 3d, we can find that the amplitude of conjugate multiplication between the CSI of two antennas also divided into two segmentations at about 5000 s due to AGC. 

As the Rayleigh fitting parameter is obtained from the amplitude of conjugate multiplication between the CSI of two antennas, when the amplitude contains the noise of AGC, the parameter is not robust to perform long-term area detection.

**Figure 4:**
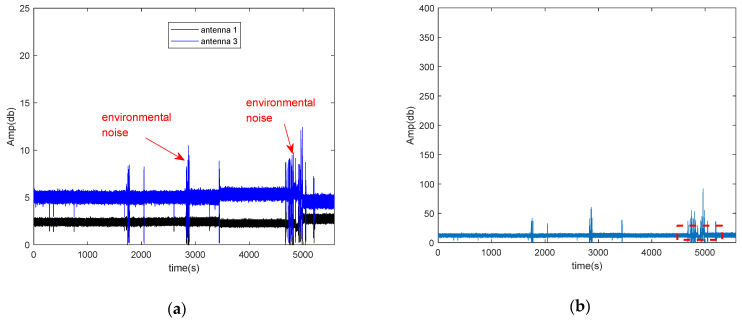
The amplitude of CSI and the conjugate multiplication between the CSI of two antennas after removing the noise of AGC. (**a**) The amplitude of CSI and conjugate multiplication between the CSI of two antennas after removing the noise of AGC in the silence environment; (**b**) the amplitude of conjugate multiplication between the CSI of two antennas after removing the noise of AGC.

The amplitude of CSI and the conjugate multiplication between the CSI of two antennas after removing the noise of AGC is shown in Figure 4. Figure 4a shows CSI amplitude of two antennas after removing the noise of AGC, and Figure 4b shows the amplitude of conjugate multiplication between the CSI of two antennas after removing the noise of AGC. From Figure 4, we can find that after removing the noise of AGC, there only exists environmental noise, and the amplitude of conjugate multiplication between the CSI of two antennas is more stable than before removing the noise of AGC.

**Figure 5:**
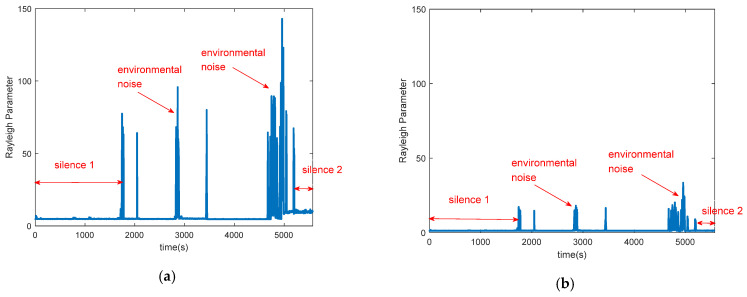
The Rayleigh fitting parameter before and after removing the noise of AGC in the silence environment (**a**) The Rayleigh fitting parameter before removing the noise of AGC; (**b**) The Rayleigh fitting parameter after removing the noise of AGC.

Since we can get more stable CSI amplitude after remove the noise of AGC, we can obtain more robust Rayleigh fitting parameter compared with Wiborder. Figure 5 shows the Rayleigh fitting parameter before and after removing the noise of AGC in the silence environment. Figure 5a shows the Rayleigh fitting parameter before removing the noise of AGC, from Figure 5a, we can find that the parameter could not remain stable (such as silence 1 and silence 2) due to environmental noise and the noise of AGC. Figure 5b shows the parameter after removing the noise of AGC, the parameter is more stable (such as silence 1 and silence 2) than Figure 5a shows.

### 4.2. The CSI Metric of Activity Intensity

In this subsection, we introduce the method for the CSI metric of activity intensity, which include how to extract Doppler frequency from the phase changes of CSI Ratio, and how to estimate activity intensity coefficient. At last, we combine Doppler frequency and activity intensity coefficient to construct the CSI metric for activity intensity estimation.

#### 4.2.1. Extracting Doppler Frequency from the Phase Changes of CSI Ratio

**Figure 6:**
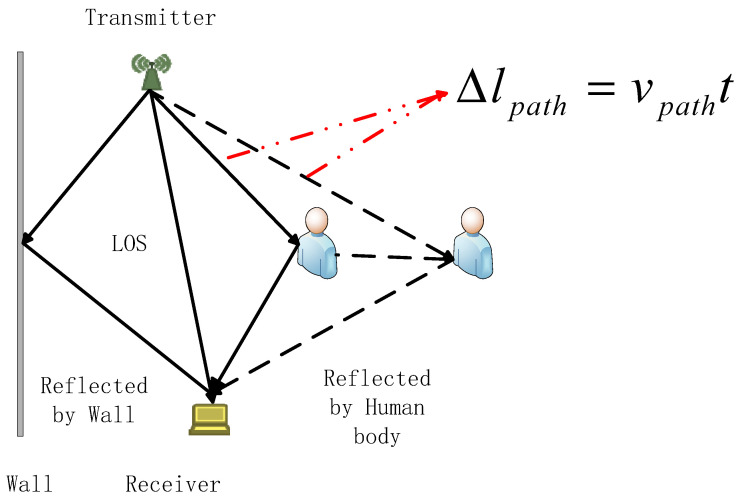
The illustration of path length change introduced by human movement.

In a typical environment with a pair of WiFi transmitter and receiver, as illustrated in Figure 6 [18]. Due to multipath effect, the signals travel through multiple paths to the receiver. The static paths are composed of the LoS path and the reflected paths from wall in the environment. Suppose there is only one dynamic path which induced by human movement, human movement can cause the path length to change, and introduce a Doppler frequency shift in the received signal [18]:

(19) $$f_{Doppler}=f\frac{v_{path}}{c},$$

where *f* is the carrier frequency of the signal, *ν_path_* is the speed of path length change, and *c* is the speed of light.

Consider only one signal, its CSI at time *t*_0_ is $H\left(f,t_{0}\right)=A_{0}e^{-j\phi_{0}}=A_{0}e^{-j2\pi f\tau_{0}}$, where *A*_0_ is the attenuation, *ϕ*_0_ is the phase of dynamic path at *t*_0_, and *τ*_0_ is the propagation delay. If the path length changes at a speed of *ν_path_*, after the short time period *t*, the path length change Δ*l_path_* = *ν_path_t*, and the propagation delay change is $\Delta \tau =\frac{\nu_{path}t}{c}$. The attenuation change can be ignored duration a short time period, the CSI of the signal is:

(20) $$H\left(f,t_{0}+t\right)=A_{0}e^{-j\phi_{1}}=A_{0}e^{-j2\pi f\left(\tau_{0}+\frac{v_{path}t}{c}\right)},$$

where *ϕ*_1_ is the phase of dynamic path at *t*_1_ The phase change Δ*ϕ* of dynamic path can be denoted as:

(21) $$\Delta \phi =\phi_{1}-\phi_{0}=2\pi f\frac{v_{path}}{c}t=2\pi f_{Doppler}t,$$

The Doppler frequency shift in the received signal can be rewritten as:

(22) $$f_{Doppler}=\frac{\Delta \phi }{2\pi t},$$

In the real environment, due to multipath-effect, the received signal is a superimposition signals from all paths. We can use Equation (22) to approximatively estimate Doppler frequency which introduced by human movements. 

**Figure 7:**
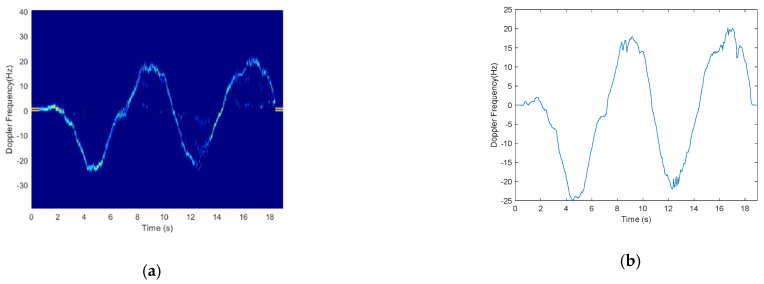
Doppler frequency extraction. (**a**) Frequency-time spectrum; (**b**) Doppler frequency.

From Equation (22), we can see that if we can measure the phase change of dynamic path, we can estimate the Doppler frequency. However, due to the random phase offset of raw CSI, the phase change cannot be directly measured from raw CSI data. Since CSI ratio can remove both amplitude noises and phase noises of raw CSI, and has higher SNR than raw CSI, and the phase change of CSI Ratio represents the path length change of reflected path. we can use CSI Ratio to obtain the phase change of dynamic path. In the complex plane, we use ${\vec{H}}_{t}$ to denote the tangent vector corresponding to dynamic component ${\vec{H}}_{d}$. We introduce a method of measuring the phase change of dynamic component through the tangent vector of dynamic component. Based on geometrical knowledge, we can conclude that the phase change of the tangent vector is equivalent to the phase change of the dynamic component, so we can measure the phase change of tangent vector to obtain the phase change of the dynamic component. We can subtract a sample point with its successor within a short time period *t* (such as 0.05 s) to calculate the tangent vector ${\vec{H}}_{t}$. The calculated result is a sequence of complex vector, and we extract the phase sequence and unwrap it to eliminate 2*π* phase jumps. Once we obtain the phase change Δ*ϕ*, we can estimate Doppler frequency approximatively using Equation (22). We employ an example to illustrate the Doppler frequency which obtained by this method. We let a person walk towards the ligature of the pair of transceivers and then to walk away twice. In theory, when the person walks towards the ligature of the pair of transceivers, the movement of the person will introduce negative Doppler frequency due to the change in radial component of the motion with respect to the receiver, and vice versa. The extracted Doppler frequency is shown in Figure 7, Figure 7a shows the Doppler frequency spectrum using this method, and Figure 7b shows the extracted Doppler frequency from Figure 7a. 

#### 4.2.2. Estimating the Activity Intensity Coefficient 

**Figure 8:**
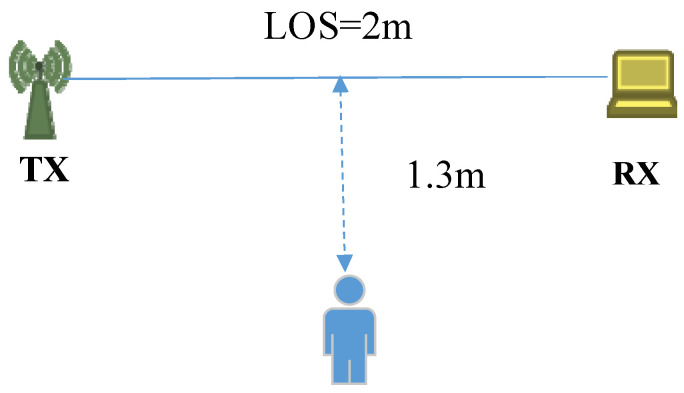
Experimental setup.

The tangent vector corresponding to the dynamic component is a complex vector, and its amplitude represents the distances between consecutive samples. In order to find the physical meaning of the sample distances, we conducted an experiment, the experimental setup is shown as Figure 8. The WiFi transceivers are placed at a distance of 2m to each other at the same height, and the position of the user is aligned with perpendicular bisector of the transceivers with a distance of 1.3 m.

**Figure 9:**
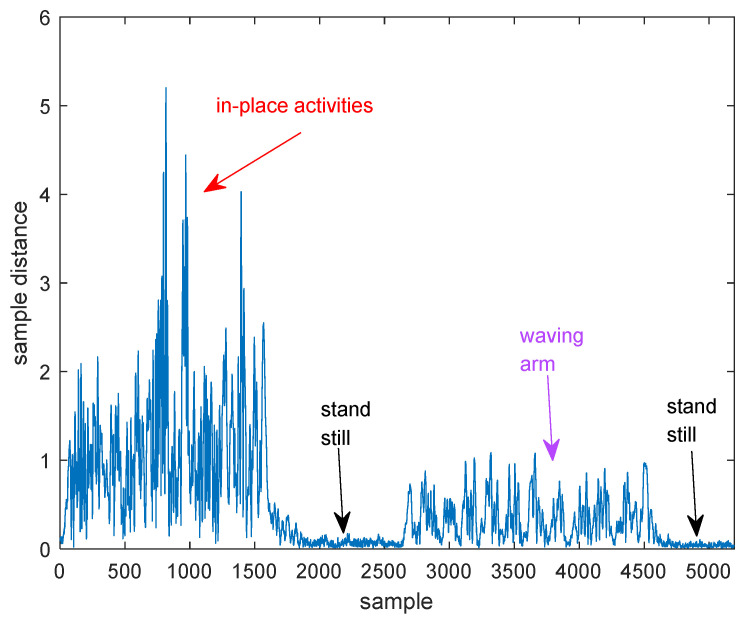
The distances between consecutive samples when the person perform two actions.

We let a person perform in-place activities at first, and then wave his arm while standing still. Figure 9 shows the distances between consecutive samples when the person doing these two actions. From Figure 9, we can find that the distances between consecutive samples are larger in case of person performing in-place activities than waving his arm. When performing in-place activities, the torso is the main reflection body part. As we all know, the torso of human body has a larger reflection area than other body parts such as arms and legs, so the signal energy reflected from torso is stronger than that reflected from arms and legs [44,45], so the amplitude of the tangent vector can represent the signal energies reflected from different body parts.

Suppose the CSI Ratio has *M* complex data samples within a short time window (such as 2 s), and the tangent vector of dynamic component also has *M* complex data samples. $\left[0,\Delta t_{2},\cdots \Delta t_{M}\right]$ is the sampling interval of each sample with respect to the first sample at *t*_0_, where Δ*t*_1_ = 0. We can construct the sample distance vector from tangent vector:

(23) $$\vec{A\left(t_{0}\right)}=\left[\alpha \left(t_{0}\right),\alpha \left(t_{0}+\Delta t_{2}\right),\cdots ,\alpha \left(t_{0}+\Delta t_{M}\right)\right],$$

where *α*(*t*_0_) is the amplitude of tangent vector data sample at time *t*_0_.

**Figure 10:**
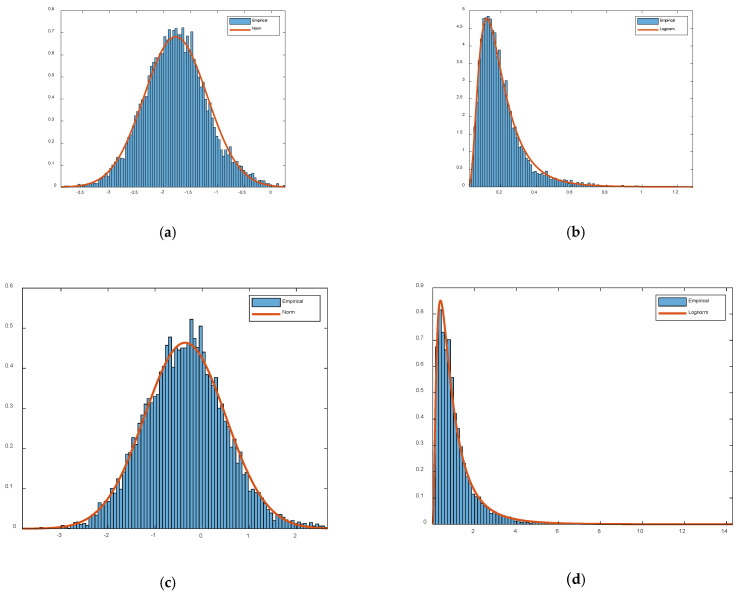
Probability Density Function (PDF) of log(*α*(*t*_0_ + Δ*t_k_*)), *α*(*t*_0_ + Δ*t_k_*) in both a silent environment and a dynamic environment. (**a**) PDF of log *α*(*t*_0_ + Δ*t_k_*) in a silent environment; (**b**) PDF of *α*(*t*_0_ + Δ*t_k_*) in a silent environment; (**c**) PDF of log *α*(*t*_0_ + Δ*t_k_*) in a dynamic environment; (**d**) PDF of *α*(*t*_0_ + Δ*t_k_*) in a dynamic environment.

Let *α*(*t*_0_ + Δ*t_k_*) represent the *k*th element of vector $\vec{A\left(t_{0}\right)}$. In order to find the statistical properties of *α*(*t*_0_ + Δ*t_k_*), we have collected 2 s tangent vector data samples generated by a silent environment and a dynamic environment (where a human performs in-situ activities), respectively. Figure 10 shows the probability density function (PDF) of log(*α*(*t*_0_ + Δ*t_k_*)) and *α*(*t*_0_ + Δ*t_k_*), respectively. From Figure 10, we see that the distribution of log *α*(*t*_0_ + Δ*t_k_*) approximately follows a normal distribution, so the distribution of *α*(*t*_0_ + Δ*t_k_*) approximately follows a log-normal distribution in both silence environment and dynamic environment.

The PDF of log-normal distribution is given by:

(24) $$p\left(\alpha \right)=\frac{1}{\sqrt{2\pi }\sigma \alpha }e^{-\frac{1}{2\sigma^{2}}{\left(\ln^{\alpha }-\mu \right)}^{2}},\;\alpha >0,$$

where *μ* and *σ* are the mean and standard deviation, respectively, of the associated normal distribution.

The geometric mean of the log-normal distribution is given by [46]:

(25) $$GM{=e}^{\mu }=e^{\frac{1}{M}\sum_{k=1}^{M}\ln^{\alpha_{k}}}={\left(e\ln^{\prod_{k=1}^{M}\alpha_{k}}\right)}^{\frac{1}{M}}={\left(\prod_{k=1}^{M}\alpha_{k}\right)}^{\frac{1}{M}},$$

We construct the activity intensity coefficient *η* using Equation (26):

(26) $$\eta =e^{GM\left(t_{0}\right)}=e^{\sqrt[{M}]{\prod_{k=1}^{M}\alpha \left(t_{0}+\Delta t_{k}\right)}},$$

Based on Equation (26), we can obtain the following two properties of *η*:

- In the silent environment, the value of *η* is very small;
- *η* is larger when activities are produced by the torso than for activities produced by the arms and legs.

#### 4.2.3. Constructing the CSI Metric for Activity Intensity Estimation

Since we have obtained the Doppler frequency and the coefficient of activity intensity, we can construct the CSI Metric for activity intensity estimation which combined with Doppler frequency and the coefficient of activity intensity. When human is still, due to the environmental noises, small Doppler frequency shifts have been observed as phase changes in CSI Ratio, and hence we can use an appropriate threshold *f_threshold_* to discriminate human motion and still states. Suppose we have obtained *N* samples of Doppler frequency sequence $\left[f_{1},f_{2},\cdots ,f_{i},\cdots f_{N}\right]$ and *N* samples of coefficient of activity intensity sequence $\left[\eta_{1},\eta_{2},\cdots ,\eta_{i},\cdots \eta_{N}\right]$ in a short time window Δ*t* (0.4 s), the fraction of time when the user is in the motion state can be denoted as:

(27) $$ratio=\frac{L\left(f_{i}>f_{threshold}\right)}{N},$$

where the numerator of Equation (27) can represent the time period when the user is non-still during a short time window. The CSI Metric for activity intensity estimation *ξ* can be constructed as:

(28) $$\xi =\sqrt{\eta_{\max}-\eta_{\min}}\times ratio=\underset{①}{\underbrace{\sqrt{\eta_{\max}-\eta_{\min}}}}\times \underset{②}{\underbrace{\frac{L\left(f_{i}>f_{threshold}\right)}{N}}},$$

where the component ① is the range of activity intensity coefficient, which can roughly quantize the fluctuation scope of activity intensity during a short time window. The component ② is the fraction of time when the user is in the motion state during a short time window. 

**Figure 11:**
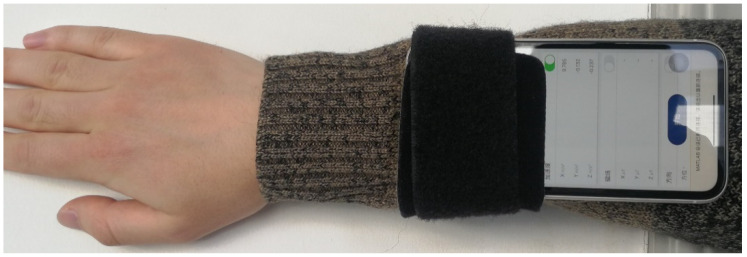
The smartphone attached to the volunteer’s arm.

In order to evaluate the effectiveness of the proposed metric, we conducted an experiment, and the experiment setup as shown in Figure 8. The volunteer first performing in-place activities, and then standing still, at last waving his arm, and repeat this procedure twice. During the experiment, a smartphone was attached to the volunteer’s arm to collect the accelerometer data, as shown in Figure 11. The build-in accelerometer of the smartphone is a tri-axial accelerometer, the magnitude of the tri-axial accelerometer data can be denoted as:

(29) $$magnitude=\sqrt{a_{x}{}^{2}+a_{y}{}^{2}+a_{z}{}^{2}},$$

where $a_{x}$, $a_{y}$ and $a_{z}$ are the three axes of the accelerometer. 

The mean of the magnitude of data can be denoted as:

(30) $$\bar{magnitude}=\frac{\sum_{i=1}^{N}magnitude_{i}}{N},$$

where *N* is the total number of data samples.

As subtracting the mean of the magnitude data can remove any constant effects such as gravity [47], we subtracted the gravitational force by subtracting the mean of the magnitude data in the following way:

(31) $$magNoG=magnitude-\bar{magnitude},$$

**Figure 12:**
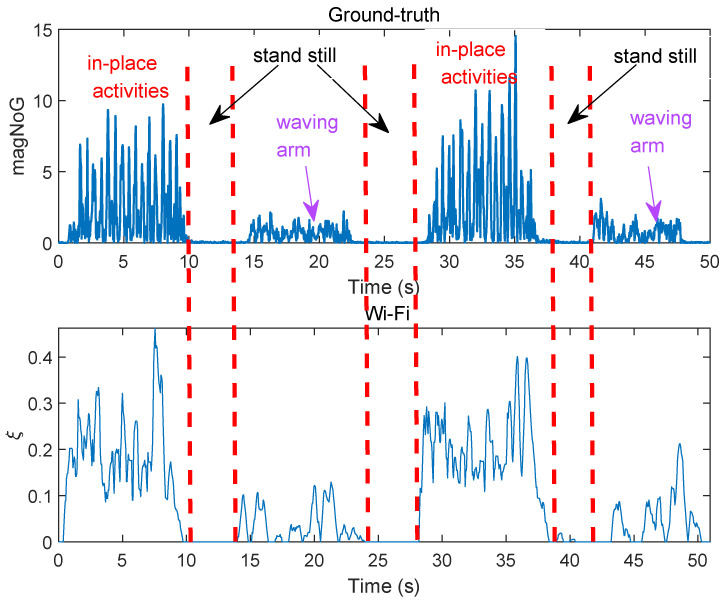
The ground-truth and the CSI metric ξ of two actions.

*magNoG* is used as the ground-truth of intensity, *magNoG* and the CSI metric for these two actions is shown in Figure 12. The upper subfigure is the ground-truth, and the bottom subfigure is the intensity estimation based on our proposed method. From the upper subfigure of Figure 12, we can see that when the user is in standing still, ground-truth is almost zero, and the ground-truth is larger when the user performs in-place activities than waving arm. From the bottom subfigure of Figure 12, we can see that our Wi-Fi based activity intensity estimation matches well the ground-truth obtained by accelerometer sensor. As shown in this experiment, the proposed CSI metric is an effective way to estimate activity intensity.

## 5. Experimental Results Analysis

In this section, we analyse the 22 days of continuous 24-h human vitality monitoring data of four volunteers which recorded by a WiMonitor system. Section 5.1 presents the experimental setup. Section 5.2 presents the evaluation for basic information of human vitality. Section 5.3 presents the statistical analysis results of 22 days continuous 24-h human vitality monitoring.

### 5.1. Environmental Setup

**Figure 13:**
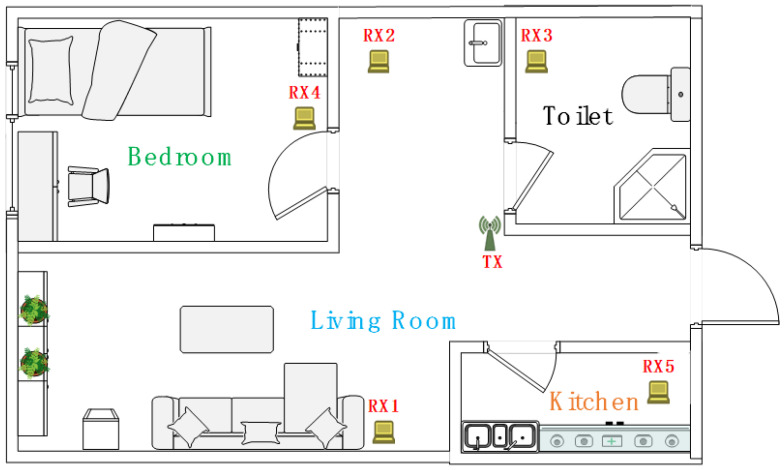
The layout of multi-room smart home environment.

**Figure 14:**
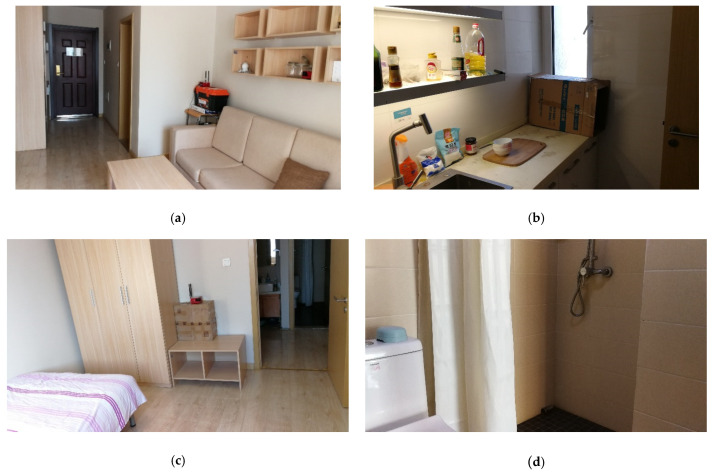
The multi-room smart home environment. (**a**) Living-room; (**b**) Kitchen; (**c**) Bedroom; (**d**) Toilet.

We employ miniPCs equipped with off-the-shelf Intel 5300 Wi-Fi cards as the transmitter and receivers. Each receiver is equipped with two antennas. The CSI tool developed by Halperin [43] is installed on each miniPC to collect the CSI samples of each received packet. The sampling rate of CSI is 200 Hz. Both the transmitter and receiver work on the 5 GHz band with a 20 MHz channel bandwidth. To evaluate the performance of WiMonitor system, we conduct experiments in a real multi-room smart home environment, which contains four subspaces: a living room, a bedroom, a kitchen and a toilet. The layout of the environment is shown in Figure 13, and the four subareas are shown as Figure 14. We employ one transmitter and five receivers to construct multiple transceivers with at least one receiver per area and placed around the corner. Four web cameras are deployed inside each area to record the ground-truth. 

We have implemented WiMonitor as a real-time system, and ask four volunteers to conduct the experiments. The basic information of these volunteers is shown in Table 1. We keep WiMonitor system running continuously, and ask the four volunteers to live alternatively in the smart home alone for 24 h, and perform the daily activities freely in their own styles (such as sleeping, eating, watching TV, etc.). We conducted a continuous 22-day experiment, which including 14 days of volunteer 1, 4 days of volunteer 2, 2 days of volunteer 3 and 2 days of volunteer 4.

### 5.2. Basic Information of Human Vitality

The basic information of vitality contains the area detection results, the motion status and activity intensity. In our experiments, at least one Wi-Fi receiver is employed in each subspace. We can choose the receiver in which the target is located in to obtain the activity intensity. For example, as illustrated in Figure 13, if the target is located in the bedroom, the system chooses RX 4 to obtain the activity intensity. If the target is located in the living-room, the system averages the activity intensities which obtained from RX 1 and RX 2.

As for area detection, we proposed a method to reduce the noise of AGC, thus can provide stable sensing boundary parameter. Based on the area detection method of Wiborder, we can achieve long-term area detection. 

In order to evaluate the performance of area detection of WiMonitor, we use two commonly used metrics precision and recall to show the performance. These two metrics are calculated by true positive (TP), false positive (FP), true negative (TN), and false negative (FN). For a certain area i, precision and recall are calculated as below:

(32) $$Precision_{i}=\frac{TP_{i}}{TP_{i}+FP_{i}},$$

(33) $$Recall_{i}=\frac{TP_{i}}{TP_{i}+FN_{i}},$$

We calculated the precision and recall for each area separately, and then take the average over all areas. Table 2 shows the precision and recall of area detection of WiMonitor over 22 days. From Table 2, we can conclude that WiMonitor can achieve high precision and recall for area detection, these results demonstrate that WiMonitor is accurate and robust for long-term monitoring.

**Figure 15:**
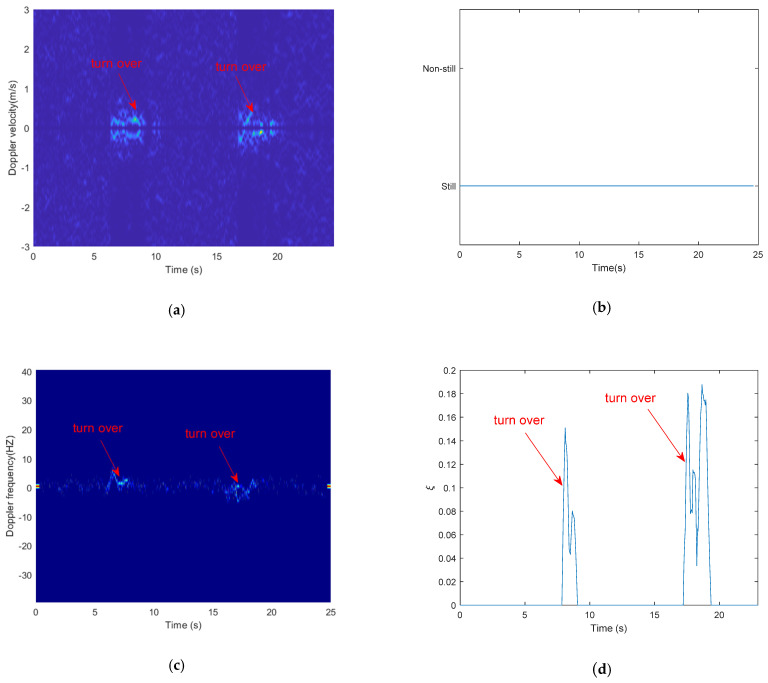
The comparison of Doppler-Music method and our proposed method for detecting turn over on the bed. (**a**) speed-time spectrum obtained from Doppler-Music method; (**b**) motion state obtained from Doppler-Music method; (**c**) Frequency-time spectrum obtained from our proposed method; (**d**) activity intensity obtained from our proposed method.

As for motion detection, WiVit [28] utilized the Doppler-Music method to extract Doppler frequency shift from conjugate multiplication between the CSI of two antennas, and used the power of Doppler frequency shift to detect human motion. It may be difficult to detect the small-scale motion actions, thus could not effectively detection such small-scale motion, as shown in Figure 15a,b. Fortunately, the proposed CSI metric for activity intensity estimation in this paper can solve this problem, and can estimate the activity intensity at the same time, as shown in Figure 15c,d.

Figure 15 shows the comparison of Doppler-Music method and our proposed method for detecting turn over on the bed. Figure 15a shows the speed-time spectrum obtained from Doppler-Music method, Figure 15c shows the frequency-time spectrum obtained from our proposed method. From Figure 15a,c, we can see that through our proposed method, we can obtain a clearer spectrum than through Doppler-Music method. Figure 15b shows the motion detection results obtained from Doppler-Music method, and as we can see, the action turning over on the bed could not be detected. Figure 15d shows the intensity of turning over, which obtained from our proposed method. 

**Figure 16:**
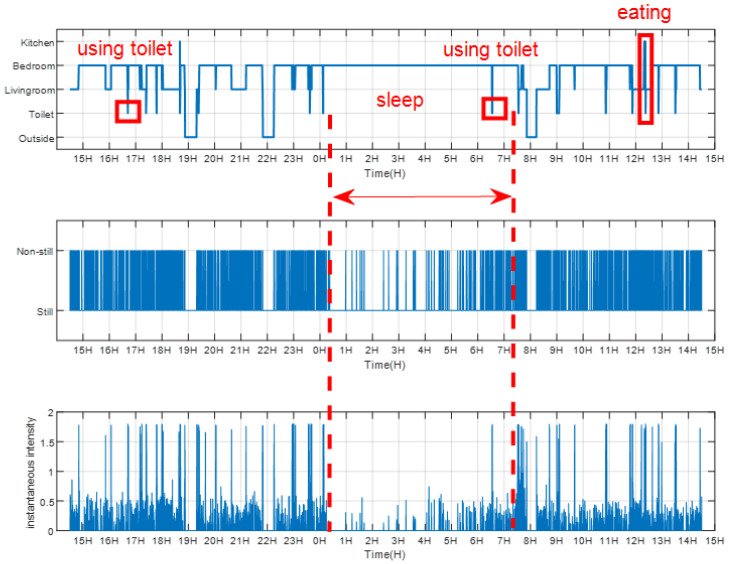
A continuous 24 h of basic information of human vitality.

A continuous 24 h of basic information of human vitality (area detection, motion detection and instantaneous activity intensity) is shown in Figure 16. The upper subfigure, middle subfigure and bottom subfigure of Figure 16 are the 24 h of area detection results, instantaneous motion state, and instantaneous activity intensity, respectively. 

Compared with Figure 16 and the corresponding ground-truth video, we can see that WiMonitor can achieve long-term vitality monitoring accurately (the markers in Figure 16 match the corresponding ground-truth).

**Figure 17:**
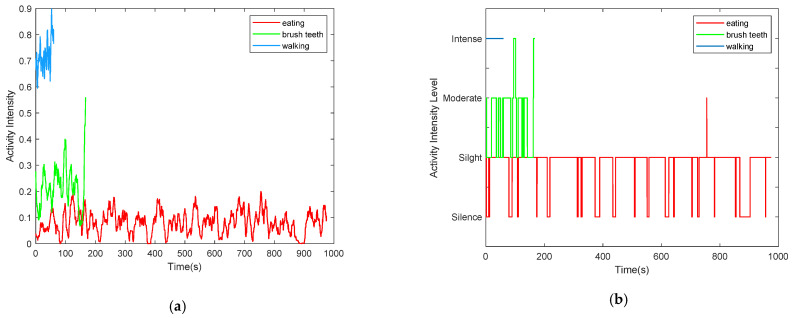
The averaged activity intensity and the corresponding activity level of the three types of daily activities. (**a**) The averaged activity intensity of the three types of daily activities; (**b**) The activity level of the three types of daily activities.

Figure 16 only shows the instantaneous vitality, in order to mine more information on human vitality, such as the characteristics of daily activities, we need to analyze vitality information from a perspective of a time period. For example, we can average the instantaneous intensity within a short time period (10 s) every 0.05 s, and category the averaged intensity into different intensity levels (such as silence, slight, moderate, and intense) according to the averaged intensity values. Take three types of daily activities (walk, brush teeth, eating) as example. the averaged activity intensity and the corresponding activity level of the three types of daily activities are shown in Figure 17. Figure 17a shows the averaged activity intensity of the three types of daily activities, and Figure 17b shows the corresponding activity level. From Figure 17, we can find that among the three activities, the activity level of walking is higher than the other two activities. 

As the WiMonitor system can continuously monitor and record human vitality information in real time, we can analyze the overall activity level of the elderly during one day or even longer to mine the daily living habits and find abnormity.

### 5.3. Statistical Analysis of 22 Days Human Vitality Information

Since WiMonitor system recorded 22 days of continuous 24 h of human vitality information data from four volunteers, in this subsection, we can analyze these data over 24-h from different perspective to capture the living habit patterns of different volunteers. 

**Figure 18:**
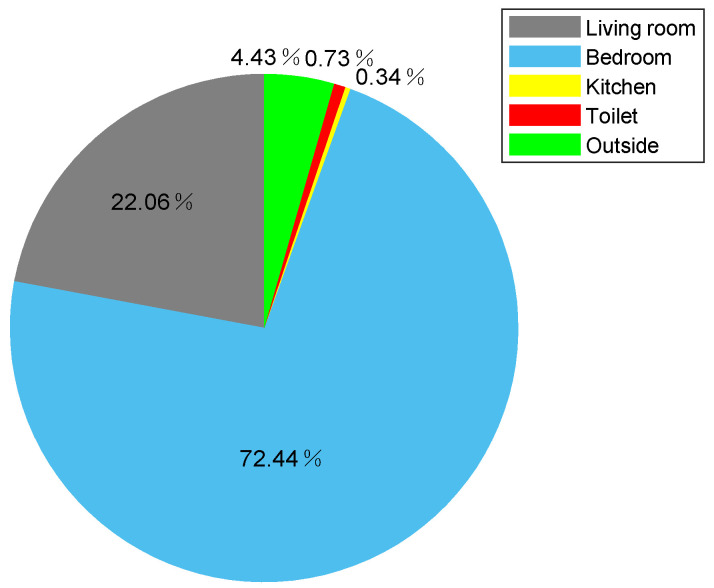
The area occupation rate of volunteer 1 during 24 h.

It is well known that in the smart home environment, physical activities are area-related, such as sleeping usually takes place in the bedroom, and cooking usually takes place in the kitchen. Analyzing the area occupation rate during 24 h of the elder, could help us to find out the living habits of the elder. Figure 18 shows the area occupation rate of volunteer 1 during 24 h. From Figure 18, we can find that the volunteer 1 spends most of the time in bedroom in one day, and spends the least time in kitchen since the volunteer 1 only enters kitchen to wash dishes after lunch and supper. 

**Figure 19:**
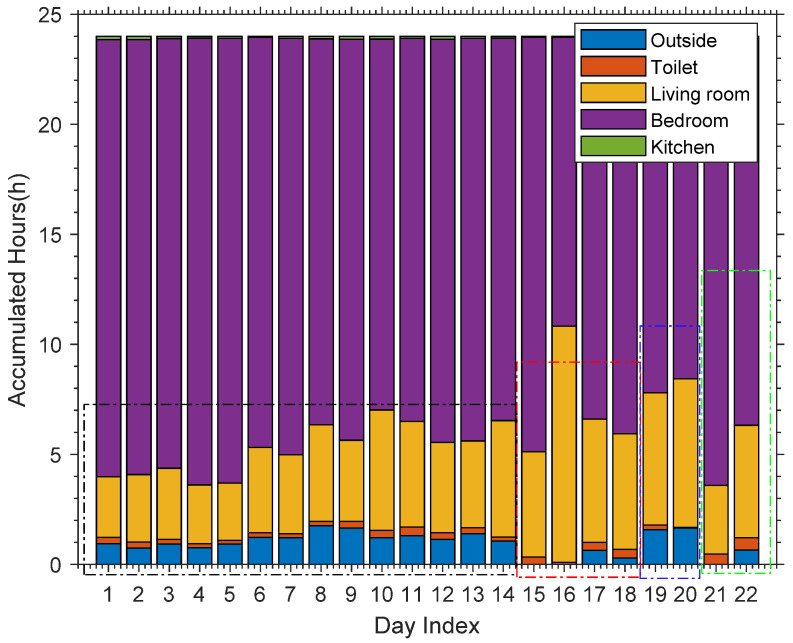
The accumulated hours of area occupation of all volunteers.

Considering different volunteers may have different living habits in the perspective of long-term area staying information, in order to find whether WiMonitor could capture the living habits of one user and the diversity of habits of different users, we can analyze the accumulated hours of area staying of all the volunteers over 22 days, as shown in Figure 19. The first 14 days are the results of volunteer 1, the fifteenth to eighteenth days are the results of volunteer 2, the nineteenth to twentieth are the results of volunteer 3 and the last two days are the results of volunteer 4. From Figure 19, we can observe the regularities in living habits of one volunteer, and the diversities of different living habits between different volunteers and the same volunteer of different days. For example, the accumulated hours of different areas from volunteer 1 are very similar during 14 days. The accumulated hours of different areas from volunteer 1 are different from volunteer 3. For volunteer 2, the accumulated hours of different areas between different days are also different. 

**Figure 20:**
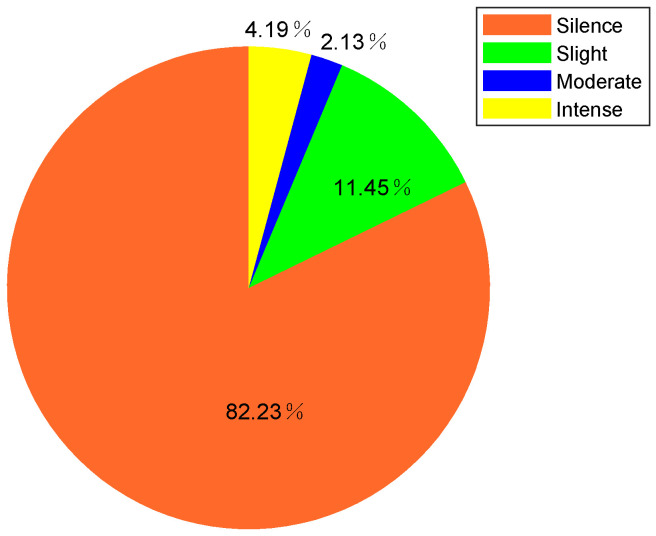
The overall intensity level rate during 24 h of volunteer 1.

**Figure 21:**
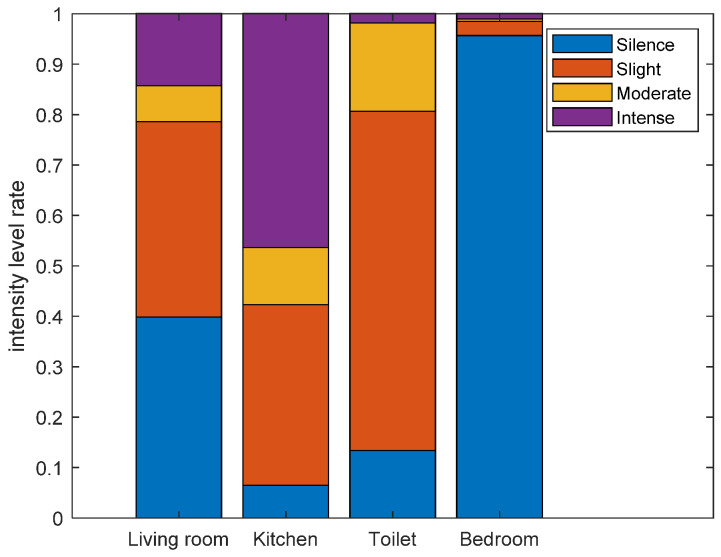
The intensity level rate of different areas during 24 h of volunteer 1.

Since activity intensity is more fine-grained sensing than motion, in this subsection, we only analyze activity intensity instead. As described in Section 5.2, we average the instantaneous intensity within a short time period (10 s), and categorized activity intensity into four levels (silence, slight, moderate, and intense) according to accumulated instantaneous intensity. Similar to long-term area staying information, we can also analyze the living habits of the volunteers in the perspective of long-term activity level information. Figure 20 shows the overall intensity level rate during 24 h of volunteer 1. From Figure 20, we can find that among the four activity levels during 24 h of volunteer 1, the rates of silence (82.23%) and slight (11.45%) level are much higher than moderate (2.13%) and intense (4.19%). Through the corresponding ground truth video, we can see that besides sleeping, the volunteer 1 spent most of the time on sedentary behaviors, such as working at a computer in the bedroom, reading book in the bedroom, and watching TV in the living room, etc. while spending less time on walking, cleaning, and exercising, etc. Considering physical activities are area-related, different types of activities usually take place in different areas, so the activity levels of different areas are also different. Figure 21 shows the activity level rate of different areas during 24 h of volunteer 1. From Figure 21, we can find that the rates of silence and slight level are higher than 90% in bedroom, due to sleeping and working at a computer are performed in the bedroom. The rate of intense level in the kitchen is the highest among the four areas, due to cleaning and wash dishes are performed in the kitchen. 

**Figure 22:**
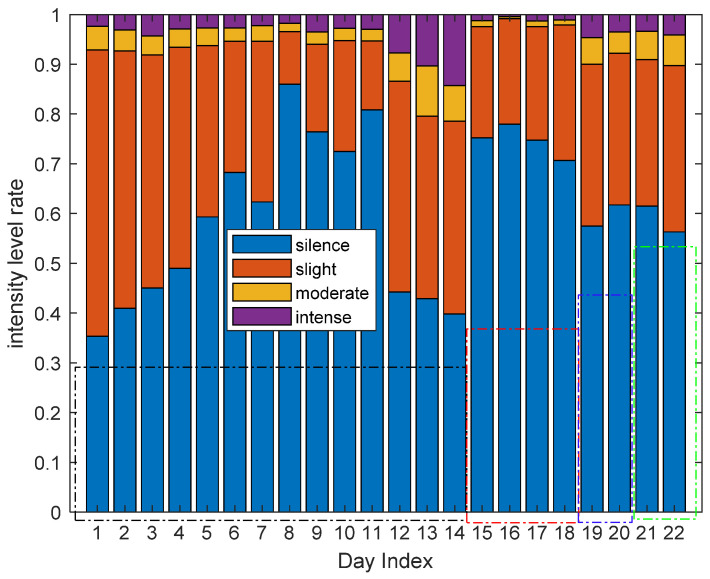
The activity level rate of all the volunteers in the living room.

In order to obtain the living habits of one user and the diversity habits of different users in the perspective of long-term activity level information, we can also analyze the activity level rate of all the 22 days of four volunteers in a specific area and in all the four areas overall. Figure 22 shows the intensity level rate of all the volunteers in the living room. Similar to Figure 19, the first 14 days are the results of volunteer 1, the fifteenth to eighteenth days are the results of volunteer 2, the nineteenth to twentieth are the results of volunteer 3 and the last two days are the results of volunteer 4. From Figure 22, we can also spot regularities in living habits of one volunteer, and the diversity of different living habits between different volunteers and the same volunteer of different days. 

**Figure 23:**
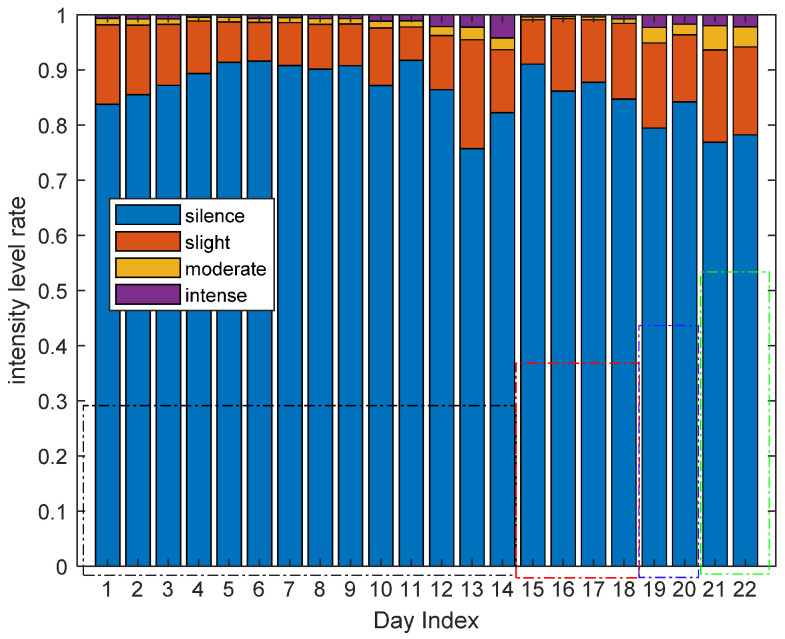
The activity level rate of all the volunteers in all areas.

Figure 23 shows the intensity level rate of all the 22 days of four volunteers in all areas overall. The first 14 days are the results of volunteer 1, the fifteenth to eighteenth days are the results of volunteer 2, the nineteenth to twentieth are the results of volunteer 3 and the last two days are the results of volunteer 4. From Figure 23, we find that all the four volunteers spend most of their time in the silence and slight activity level, which could be considered as a sedentary lifestyle. 

**Figure 24:**
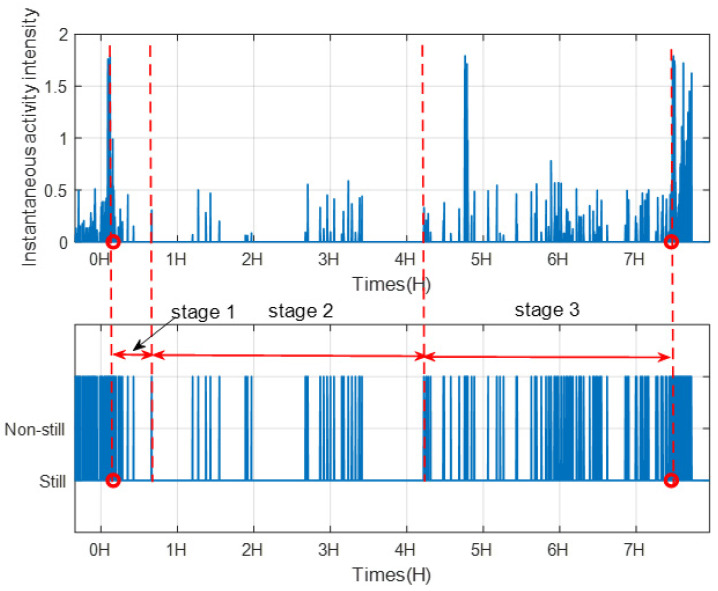
The instantaneous activity intensity and the motion status during sleep of volunteer 1.

Through analysis statistical properties of human vitality information, we not only can capture the living habits of users, but also detect abnormality. Take sleep as an example, since sleep plays an important role in our health and well-being. Low quality of sleep will increase the risk of sleep disorders. In this paper, we can use instantaneous activity intensity to analysis body movements during sleep. We consider motion states as those instantaneous activity intensity values which are larger than zero. Figure 24 shows the instantaneous activity intensity and the corresponding motion status during sleep of volunteer 1. From Figure 24, we can find that the body movement during sleep can be roughly divided into three stages: stage 1, stage 2 and stage 3. The body movement is frequent in stage 1, as the volunteer 1 is going to sleep. The body movement is less frequent in stage 2 and is the most frequent in stage 3. 

**Figure 25:**
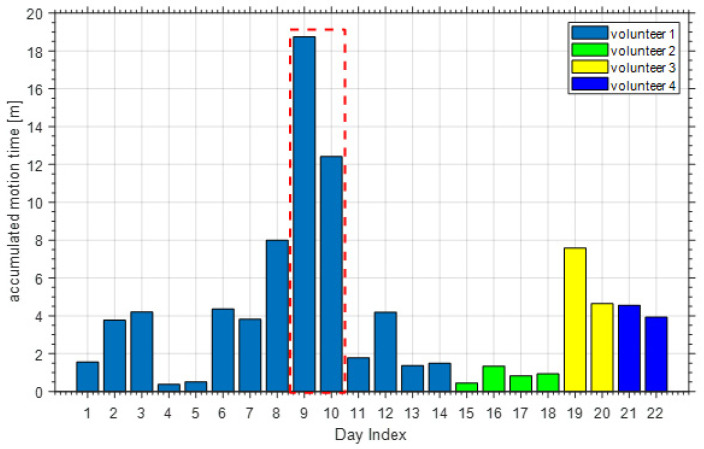
The accumulated motion time during sleep of all the volunteers.

For an individual subject, it is true that the body movement during sleep over one night cannot tell if the subject sleeps well or not, however, the body movement pattern over weeks or months could be a good indicator for one’s sleep quality (especially for very abnormal patterns). In this paper, we used the accumulated body movement time during sleep to represent the body movement frequency. Figure 25 shows the accumulated motion time during sleep of all the volunteers, the first 14 days are the accumulated motion time of volunteer 1. Only consider volunteer 1, we can find that during the 14 days, the accumulated motion time in the ninth and tenth day are much longer than the other twelve days. Through compared with the corresponding ground-truth video, we can find that the volunteer 1 suffered from insomnia for some reason in those two nights.

## 6. Discussion

In recent years, many device-free indoor localization systems have been proposed, and the existing device-free indoor localization systems can be categorized as fingerprint-based and geometric mapping-based. Many methodologies have been proposed to improve the performance, such as deep learning and transfer learning [48,49], etc. Wang et al. [48] presented a deep learning based indoor fingerprinting system DeepFi, the DeepFi architecture has four hidden layers, and a probabilistic data fusion method is developed for online localization. The mean localization error of DeepFi is about 0.9 m. Rao et al. [49] proposed DFPWL system which used transfer deep learning method to cope with time-varying characteristic of CSI which caused by environment changes, the mean localization error about 1.1 m is achieved in [49]. Unlike fingerprint-based method, geometric mapping-based method does not need to build fingerprint database. MaTrack [16] employ the angle-of-arrival (AoA) information to locate the user. The median localization accuracy of MaTrack is about 0.6 m. SiFi [19] uses a single AP to locate the target. The median localization accuracy is about 0.93 m. 

Though the above two types of localization approaches are very useful in many applications, such as indoor navigation, augmented reality, disaster rescue, etc. However, in a typical home environment, the rooms are usually divided by walls, detecting which room a person is staying is essential for daily life monitoring. This requires the system to detect the precise sensing boundary between different rooms. However, the above two types of localization approaches couldn’t fulfill this requirement, because 20 cm localization error could make wrong judgement of room-level localization when a person is close to the room door. Different from the above works, our proposed approach uses a novel CSI metric for through-wall discrimination to determine the precise sensing boundary between different rooms, thus can achieve accurate room-level detection (the *precision* and *recall* of our approach are both higher than 96%).

According to [50], on average, there are already more than eight Wi-Fi-enabled devices in a typical U.S. home environment. In China, with the rapid development of the Internet of Things, Wi-Fi devices are ubiquitous in home environments, ranging from Wi-Fi routers and mobile phones to Wi-Fi-enabled home appliances (e.g., TVs, refrigerators). We strongly believe in the near future these Wi-Fi enabled devices could be utilized for contactless sensing without additional cost, enabling various sensing applications in a home setting, such as fall detection, respiratory monitoring, continuously daily activity monitoring (if Wi-Fi is outdated, there will be Wi-Fi like wireless signals such as 5G/6G).

## 7. Conclusions

In this paper, we propose a continuous long-term human vitality sensing system called WiMonitor using commodity Wi-Fi devices. For an elder who lives alone, the WiMonitor system could capture vitality information continuously in real-time without any human effort on offline-training. WiMonitor could achieve continuous long-term accurate area detection by removing the noise of AGC. Moreover, we construct a CSI metric for estimate the instantaneous activity intensity real time. We recruit 4 volunteers to conduct a continuous 22-days experiment, and record the long-term vitality data. Through analysis, we can conclude that the recorded long-term vitality information can be used to obtain the routine habits of the user and detect any abnormality. As the next step, we are going to use our system to track vitality information over a longer term, with the hope that we could observe the long-term routine change trends using the data from several months. We envision our WiMonitor system could be a useful system to provide abundant datasets of the daily living of the elderly to help not just researchers but also healthcare personnel.

## Figures and Tables

**Table 1:** The basic information of the volunteers.

Volunteer ID	Gender	Age	Height (cm)	Weight (kg)
1	Male	34	173	70
2	Male	29	175	68
3	Male	24	170	66
4	Male	24	172	60

**Table 2:** *Precision* and *Recall* of area detection of WiMonitor over 22 days.

**Day ID**	**1**	**2**	**3**	**4**	**5**	**6**	**7**	**8**	**9**	**10**	**11**
Precision [%]	98.5	97.7	97.6	97.5	98.2	97.2	96.7	97.8	97.1	96.9	99.3
Recall [%]	97.3	99.0	97.2	98.9	97.0	98.7	98.3	97.0	98.4	98.6	99.0
**Day ID**	**12**	**13**	**14**	**15**	**16**	**17**	**18**	**19**	**20**	**21**	**22**
Precision [%]	98.6	97.1	98.0	97.0	97.1	97.6	97.5	96.9	97.2	96.3	97.5
Recall [%]	96.7	98.2	96.8	97.3	98.1	98.7	98.4	97.2	98.5	98.6	99.5

## Data Availability

Not applicable.
